# Red blood cell rheology during a complete blood count: A proof of concept

**DOI:** 10.1371/journal.pone.0280952

**Published:** 2023-01-27

**Authors:** Pierre Taraconat, Jean-Philippe Gineys, Damien Isebe, Franck Nicoud, Simon Mendez

**Affiliations:** 1 Horiba Medical, Montpellier, France; 2 Institut Montpellierain Alexander Grothendieck, CNRS, Univ. Montpellier, Montpellier, France; Université Claude Bernard Lyon 1, FRANCE

## Abstract

Counting and sizing blood cells in hematological analyzers is achieved using the Coulter principle. The cells flow in a micro-aperture in which a strong electrical field is imposed, so that an electrical perturbation, called pulse, is measured each time a cell crosses the orifice. The pulses are expected to contain information on the shape and deformability of Red Blood Cells (RBCs), since recent studies state that RBCs rotate and deform in the micro-orifice. By implementing a dedicated numerical model, the present study sheds light on a variety of cells dynamics, which leads to different associated pulse signatures. Furthermore, simulations provide new insights on how RBCs shapes and mechanical properties affect the measured signals. Those numerical observations are confirmed by experimental assays. Finally, specific features are introduced for assessing the most relevant characteristics from the various pulse signatures and shown to highlight RBCs alterations induced by drugs. In summary, this study paves the way to a characterization of RBC rheology by routine hematological instruments.

## 1 Introduction

In humans, oxygen supply to the tissues is achieved by Red Blood Cells (RBC), through the circulatory system. Indeed, RBCs are enucleated cells that are rich in hemoglobin, a protein able to bind with oxygen. Anemia characterizes a disfunction in the oxygen delivery, which may occur in case of blood loss, impaired production, or increased destruction of erythrocytes. Such disorders are diagnosed by performing a Complete Blood Count (CBC), a routine clinical analysis. Among the parameters rendered in a CBC, one may find the mean volume of the erythrocytes, called the mean corpuscular volume (MCV), the RBC volume distribution width (RDW), the hemoglobin concentration (Hb) and the RBCs count. These are hematological parameters used for detecting and monitoring different types of anemia [1, 2]. Furthermore, the hematological parameters associated to RBCs (in particular RDW) were shown to be significantly impacted in various pathological conditions [3–5], mainly in cardiovascular diseases.

Most of the gas exchanges occur in the micro-capillaries, whose diameters are typically smaller than those of RBCs. Indeed, a typical size of erythrocytes is 8 μm, while capillaries can be smaller than 5 μm in diameter. Hence, the deformability of RBCs is a key feature for them to flow inside the circulatory system. The capability of RBCs to deform relies on the viscoelastic properties of their membrane, their content and the reduced volume of the cell. The reduced volume is the ratio of the cell volume with the volume of the sphere having the same surface area than the cell membrane. In normal conditions, erythrocytes present a discocyte shape [6] and have a reduced volume typically around 0.65. This means that RBCs are deflated, which significantly contributes to their capability to deform. Several pathologies have been associated to impaired RBCs shape and/or deformability [7] such as diabetes [8–10], sickle cell disease [11–14], malaria [15–17], COVID-19 [18, 19], and hereditary spherocytosis [20, 21]. In this respect, several methods have been introduced over the years for measuring the RBCs deformability [22–30]. To our knowledge, only ektacytometry [31–34], is commercially available, although it is not used routinely. Providing an erythrocytes deformability parameter alongside the CBC would allow a finer analysis of the RBCs population. In this paper, we are interested in assessing how existing systems used in hematology may be enhanced to provide a more complete characterization of RBCs. While hematology analyzers feature a constriction, which is classical for analyzing cells deformability in extensional flow [35–38], it is actually the shear region of the flow that is used in the following to infer RBCs properties. Furthermore, it is worth mentioning that thousands of cells are analyzed each second in commercial hematological analyzers, which would thus provide high-throughput deformability analysis, directly in routine instruments.

**Fig 1:**
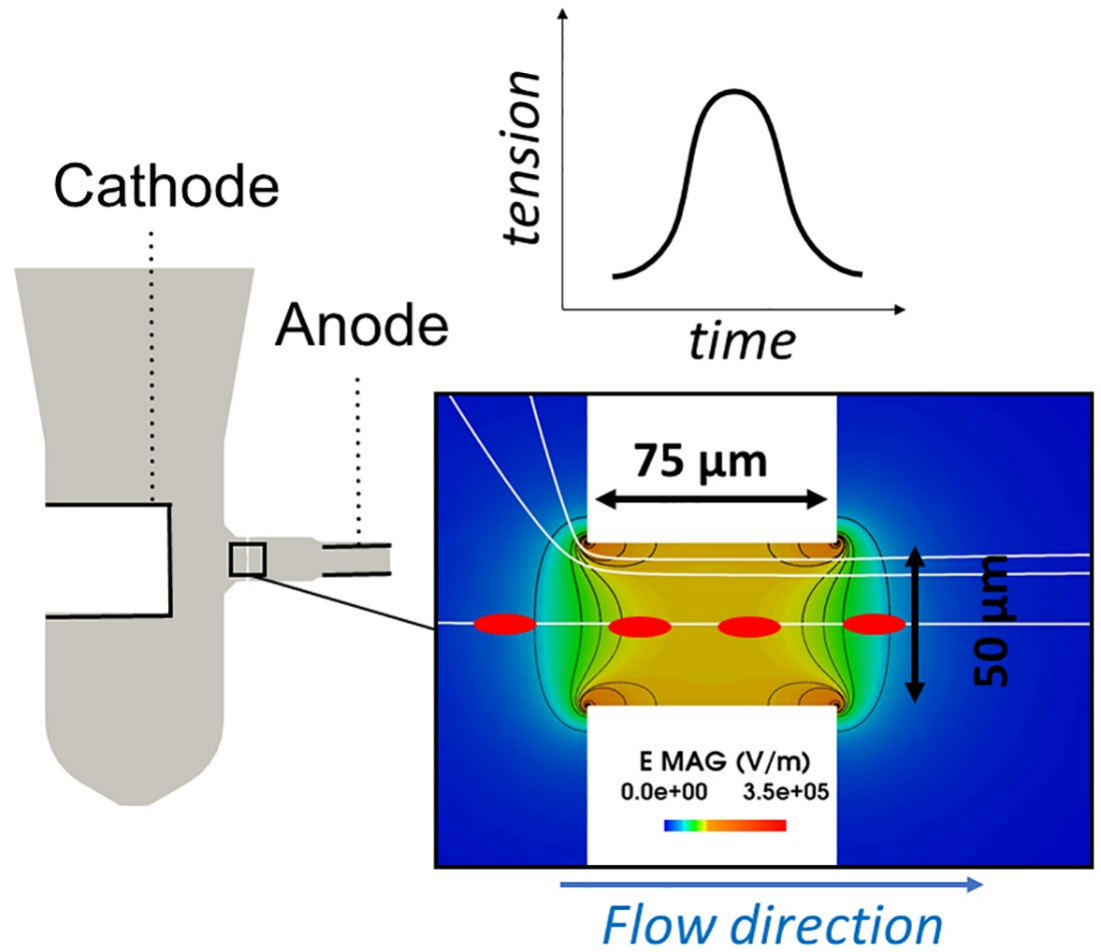
Impedance system based on the Coulter principle to count and size particles: The left image is a slice through the complete domain; the color image shows the map of electrical field in the aperture, the red ellipses representing a time lapse of an RBC flowing through the sensor, with the associated tension pulse along time depicted above. It should be noted that the constriction depicted in the color picture is cylindrical, the RBC flowing over the symmetry axis.

In hematological instruments, the RBC count, MCV and RDW, are often performed in an impedance system, based on the Coulter principle [39]. To do so, blood is first diluted, then flows through a sensor that involves a cylindrical micro-orifice (or aperture) and two electrodes located across the orifice (see Fig 1). The micro-orifices are typically 75 μm long and 50 μm in diameter [40–44]. The electrodes, powered with a constant intensity current, generate an electrical field in the flowing suspension. Because of the constriction, the electrical field is only intense inside and close to the orifice: this is the detection area. The blood cells having a conductivity significantly lower than the suspending medium, their passage through the micro-aperture results in an increase of resistivity, which in turn leads to a tension pulse measurable at the electrodes, as Fig 1 illustrates. As simultaneous passages are rare due to the large dilution rate employed, the pulses count directly leads the cell count in the samples, while for each pulse, the amplitude Δ*U* is assumed to be proportional to the cell volume *V*_*p*_ [43]:

$$\begin{array}{c} \Delta U\propto f_{s}E^{2}V_{p} \end{array}$$ (1)

with *E* the electrical field magnitude and *f*_*s*_ a shape factor. Indeed, two particles of same volume *V*_*p*_ may disturb the electrical field differently, depending on their shape and orientation with respect to the electrical field. The influence of particle shape and orientation on the pulse, for a given volume, is accounted by introducing the shape factor *f*_*s*_. For instance, the shape factor of a rigid RBC varies between 1.2 and 2.9 depending on its orientation with respect to the electrical field, while the shape factor of a sphere is 1.5, as theoretical developments have shown [45, 46]. Note that the shape factor of an infinitely elongated cell aligned with the electrical field is 1, the smallest possible value for *f*_*s*_. To calculate the RBC parameters of the CBC (viz. the MCV and the RDW), the volume histogram [44, 47], is built from the amplitudes of a large number of pulses to guarantee statistical convergence. In the following, we focus on RBCs only, which are easy to isolate from the other blood cells due to the difference in volume.

The linearity between the signal amplitude and the particle volume requires that the electrical field is homogeneous in the detection area and that all RBCs have the same shape factor. This is not the case for near-wall trajectories of RBCs. First, the electrical field is known to be highly heterogeneous near the orifice edges [42, 46, 47], as illustrated in Fig 1. Besides, next to the aperture walls, high shear stresses result in rotations [42–44] and deformations [42] of RBCs. These dynamical edge-effects lead to variations of *f*_*s*_ while the RBC crosses the detection area, thus invalidating the linearity assumption between Δ*U* and *V*_*p*_. This explains why many efforts have been made for detecting and rejecting pulses impaired by the edge-effects [43, 45, 48], or for designing Coulter counters with hydrofocusing [49] to prevent RBC from passing next to the orifice wall.

However, although they are unsuited for the volume measurements, the pulses impacted by dynamical edge-effects (in particular due to the high-shear flow near the wall) may provide information on the shape and deformability of the cells. Indeed, previous studies suggest that inferring particles shapes from the impedance pulses is possible. Golibersuch [50, 51], demonstrated the possibility to quantify the asphericity of particles flowing in long apertures (about 20 μm diameter and 290 μm length): due to multiple rotations in the orifice, the shape factor changes, and variations increase with the degree of asphericity of the particles. Kachel [47] and Grover [52, 53], also reported significant differences between pulses originating from RBCs, spheres and spheroids. In a recent study, we have focused on RBCs and shown that the shape factor variation of normal RBCs during a rotation is close to 0.5, whereas 1.7 is expected for rigid discocytes [42]: consequently, the RBC deformability impacts the measured signal, but more advanced studies are needed to quantify the relation between RBC shape and rheological properties and impedance pulses.

In order to assess the effect of morpho-mechanical characteristics of RBCs on impedance measurements, we mainly rely on numerical simulations. Computational techniques have the advantage of circumventing the metrology difficulties (high-throughput experiments, speeds of the order of a few meters per second), but also allow a perfect control of the characteristics of the RBCs, which is impossible to manage in an experiment. However, validation against experimental data is indispensable to assess the relevance of the simulations in such complex systems. This study thus presents a series of numerical simulations that shed light on a variety of RBCs dynamics in Coulter-based systems and explains how dynamics affects the measured pulses (Sec 3). In addition, the impact of the RBC properties on the measured pulse is investigated with a numerical approach (Sec. 4). Comparisons with experimental data are shown, in order to validate the numerical findings. Then, based on the numerical observations, original features (or metrics) are introduced for assessing the most relevant pulse patterns, which are expected to be future markers of pathological states. Finally, in Sec. 5, measurements from drug-treated cells are presented and the metrics are shown to highlight RBCs disorders of morpho-mechanical nature (viz. sphericity and rigidity).

## 2 Materials and methods

### 2.1 Numerical simulations

**Fig 2:**
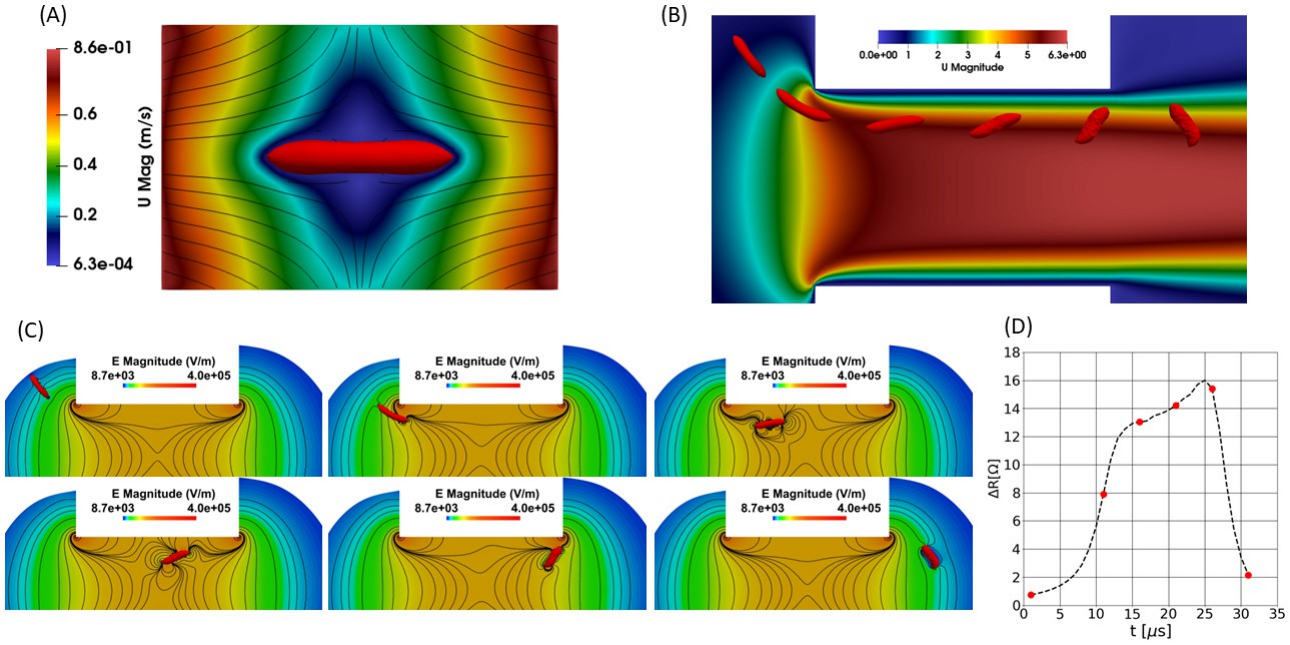
Numerical pipeline for the simulation of the RBC dynamics and of the associated electrical pulse in a Coulter counter. (A) Simulation of the RBC elongation in an extensional flow, which reproduces the deformations occurring before the cell enters in the aperture. Proper boundary conditions are applied to reproduce the extensional flow seen by the RBC along its trajectory. The typical extensional rate in picture A is 5104 s^−1^. (B) Sequence of RBC shapes during the simulation of the RBC dynamics in the aperture, shown over the velocity field (without cell). The elongated cell depicted in A is used as the initial state of the RBC in the simulation shown in B. (C) Electrostatic simulations performed for each RBC shape issued from B. (D) Resistive pulse obtained by gathering the results of the electrostatic simulations of picture C.

Simulating the dynamics of deformable particles in impedance systems is challenging. It is a fluid-structure interaction problem, where the flow inside the RBC and outside the RBC need to be predicted and coupled to the membrane mechanics. On the one hand, RBCs deform before entering the sensing region, so that the computational domain cannot be restricted to the micro-orifice. On the other hand, simulating the RBCs dynamics in the entire domain would induce prohibitive computational costs. Therefore, a specific series of simulations, described in Fig 2, is required for handling the calculation of the RBC dynamics [42, 43]. Details and validation of this simulation pipeline have been presented by Taraconat et al. [42]. In a nutshell, the dynamics of the RBC is calculated in two steps. In the first step, illustrated in Fig 2A, the RBC is elongated in an extensional flow that reproduces the deformation occurring before the orifice. From a simulation of the flow (without cells) in the whole domain, velocity gradients are extracted along the cell trajectories. A small domain centered on an RBC applies a time-varying purely extensional flow as if we followed the cell along its trajectory. The second step is dedicated to the calculation of the cell dynamics in the micro-orifice (see Fig 2B). The elongated RBC resulting from the first simulation is used as the initial state of the RBC in the second computation. This second simulation is performed in a domain restricted to the sensing region, which allows a substantial reduction of the computational cost. The electrical pulse associated to the RBC dynamics inside the aperture is calculated separately. A first calculation of the electrical field is performed without cell. Then, for each of a time series of position and deformation of the RBC predicted by the dynamic simulation (see Fig 2B), an electrostatic simulation is performed to compute the impedance of the system perturbed by the cell passage, as shown in Fig 2C. Thanks to the electrostatic assumption, dynamic and electrical effects are decoupled, and the electrical field is obtained by solving the Laplace equation with an insulating condition on the RBC membrane [42]. A time series of resistive perturbations associated with the presence of the RBC in the micro-orifice is thus obtained, which is a function of the RBC dynamics (see Fig 2D).

The different simulations are performed with the YALES2BIO solver (https://imag.umontpellier.fr/ yales2bio/). In YALES2BIO, the RBC dynamics is calculated by solving the fluid-structure Interaction problem between the RBC membrane and the fluids located inside and outside of the membrane [54–57]. The fluids are modeled according to Navier-Stokes equations, while the membrane is modeled with both Skalak [58] and Helfrich [59] laws (see Eqs 2 and 3, below), which account for area, shear and bending resistance of the membrane.

$$\begin{array}{c} W_{sk}=\frac{G_{s}}{2}\left[{\left(\lambda_{1}^{2}+\lambda_{2}^{2}-2\right)}^{2}+2\left(\lambda_{1}^{2}+\lambda_{2}^{2}-\lambda_{1}^{2}\lambda_{2}^{2}-1\right)\right]+\frac{E_{a}}{4}{\left(\lambda_{1}^{2}\lambda_{2}^{2}-1\right)}^{2} \end{array}$$ (2)

$$\begin{array}{c} \epsilon_{b}=\frac{E_{b}}{2}\int {\left(2H-c_{0}\right)}^{2}dS \end{array}$$ (3)

*G*_*s*_ and *E*_*a*_ in Eq 2 denote the shear modulus and the area modulus, respectively, while λ_1_ and λ_2_ are the principal values of strain in the membrane plane. *W*_*sk*_ is the elastic energy density in the membrane. In Eq 3, *ϵ*_*b*_ is the curvature energy of the whole membrane. *E*_*b*_, *H* and *c*_0_ refer to the bending modulus, the mean curvature and the spontaneous curvature (set to 0 in our simulations), respectively. The coupling of the membrane with fluids equations is allowed by the Immersed Boundary Method [60].

The simulations are performed in a configuration consistent with the RBCs analysis unit of the ABX Micros 60 (one of the blood analyzers commercialized for CBCs by HORIBA Medical): a pressure drop of 200 mbar sustains the flow and the aperture is 50 μm diameter with a length of 75 μm. The meshes and fluid domains used for these simulations are similar to those employed in [42, 43]. The RBC membrane is discretized with triangular meshes whose typical size is 0.3 μm. Furthermore, the fluid meshes are refined to 0.3 μm around the cell trajectory. This is required for ensuring a proper coupling between the fluids and the membrane all along the RBC path in the micro-orifice.

### 2.2 Experimental acquisitions

This study involves human peripheral blood samples. We have not sought for an institutional review board (ethics committee) approval because we acquired samples in the framework of a supply contract with CHU de Montpellier, which put in place provisions for the use of the samples for research purposes. More precisely, samples are collected as part of standard care and provided anonymously to HORIBA Medical. Patients were informed that their residual blood samples would be used for research purposes and did not express opposition. In this contract, all parties declare to conform with the French regulation in terms of sample disposal, that ensures the respect for dignity, integrity, and non-ownership of the human body. The experiments were performed from blood samples withdrawn from healthy patients in K3EDTA tubes (VACUETTE) and analyzed within the 6 hours after withdrawal. Experimental signals were recorded from an ABX Micros 60 (HORIBA Medical). When the sample tube is introduced in the instrument, a needle withdraws an aliquot from the tube and distributes the collected volume to the different units of the automaton. In this work, we focus exclusively on the RBCs counting chamber, which is the simulated device. In the RBCs chamber, the sample is diluted by a factor 1/15000 in the ABX Minidil LMG (HORIBA Medical) electrolytic reagent. Then, a vacuum pump aspirates the suspension through the micro-orifice, while the constant direct current is applied by two electrodes. During the analysis, the terminal voltage is amplified by the ABX Micros 60 hardware system and given as an input of an in-house LabVIEW^*TM*^ (National Instruments) code, dedicated to the recording of the electrical pulses only [42, 43] (viz. without the baseline). The instrument used in these experiments differs from the commercial version of the ABX Micros 60 in the sense that the bandwidth has been increased to 150 kHz. This is done to alleviate the signal distortion induced by the electronical system implemented in the commercial version.

## 3 Impact of RBCs dynamics on the measured signals

### 3.1 Simulations over different trajectories

From a given RBC, simulations were performed for different trajectories inside the micro-orifice (from the center to vicinity of the aperture edges). The RBC is parametrized by area (*E*_*a*_), shear (*G*_*s*_) and bending (*E*_*a*_) moduli of 2.5 × 10^−1^ N.m^−1^, 2.5 × 10^−6^ N.m^−1^ and 6.0× 10^−19^J, respectively (see Eqs 2 and 3). The RBC has a volume *V*_*p*_ of 93 μm^3^, and its reduced volume Q is 0.65 (Q is the ratio between *V*_*p*_ and the volume of the sphere having the same surface area as the membrane). Note that these parameters are relevant for normal (viz. healthy) RBCs [61, 62]. The suspending fluid is mostly water, thus a kinematic viscosity *ν*_*ext*_ of 1.0 × 10^−6^ m^2^.s^−1^ and a density *ρ* = 1000 kg.m^−3^ are set in the simulations. Besides, the conductivity *σ*_*ext*_ of the suspending medium is 2.27 S.m^−1^, which is typical of the electrolytes used in industrial systems [40, 42, 43]. Concerning the fluid inside the membrane, a kinematic viscosity *ν*_*in*_ equaling 18.0 × 10^−6^ m^2^.s^−1^ was set to conform with the 20°room temperature of the experiments [62]. The internal conductivity *σ*_*in*_ is set to zero to mimic a perfectly insulating cell, as discussed in Sec. 2.

**Fig 3:**
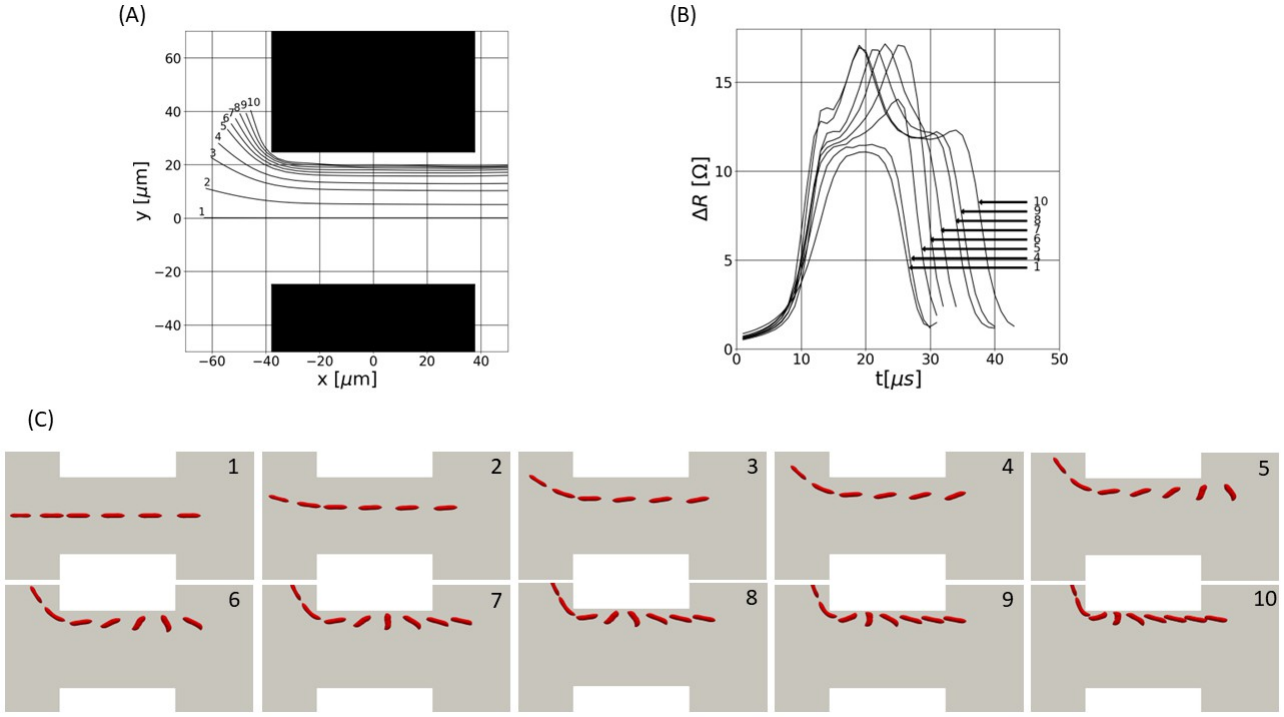
Numerical simulations of the RBCs dynamics and of the associated electrical pulses for different trajectories: (A) RBC trajectories inside the aperture (the aperture center is located at the origin of the coordinate system); (B) Resistive pulses; (C) RBCs dynamics in the aperture. In picture B, cases 2 and 3 are not shown since no substantial variations are found between cases 1 and 4, in terms of pulse profiles. In all pictures of C, the first two RBC shapes are separated by 11 μs. The remaining shapes are displayed at 4 μs intervals.

The simulated trajectories, the electrical responses and the RBC dynamics are shown in Fig 3A–3C, respectively. Signatures related to centered paths are ‘bell-shaped’, as cases 1–4 show. In contrast, cases 5–10 highlight the variety and the complexity of the pulses generated by RBCs flowing near the orifice wall. In addition, the closer the particle path to the aperture edges, the longer the pulse duration (see Fig 3A and 3B). Indeed, an RBC spends more time in the aperture when it follows a near-wall trajectory, as compared with a central path, due to the smaller velocity values near the walls.

**Fig 4:**
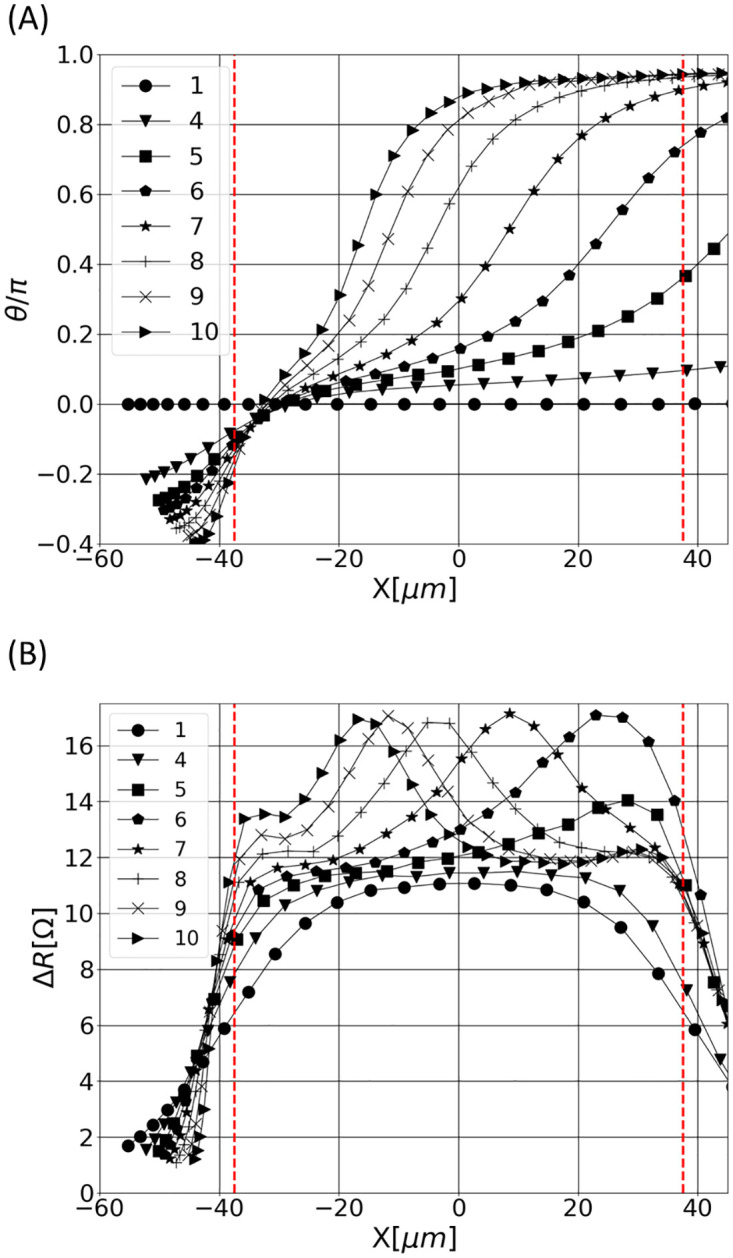
RBC orientations and resistance pulses as a function of the cell location inside the aperture, for different trajectories: (A) RBC orientation (is zero when the particle is aligned with the aperture axis); (B) Resistive pulses.

Evolutions of the RBC orientation *θ* as a function of the RBC longitudinal location in the aperture (noted *x*) are shown in Fig 4A, for different trajectories of Fig 3A. Given an RBC shape inside the aperture (see Fig 3C), the Inertia Equivalent Ellipsoid (IEE) is calculated [42], to characterize the shape and orientation of the RBC: it is the ellipsoid having the same inertia tensor as the RBC. Then, *θ* is defined as the angle between the IEE main (larger) axis and the aperture axis. For the most centered paths, the cell is aligned with the aperture axis (*θ* ≈ 0) during its entire path in the sensing region, as depicted by cases 1–4 in Figs 3C and 4A. This is explained by the flat velocity profile at the center region of the orifice. In contrast, high shear levels in the wall vicinity make the RBC rotate (see cases 5–10 in Figs 3C and 4A). In particular, the RBC performs a quarter turn in case 5, while it achieves almost a half-turn for trajectories closer to the aperture wall (see cases 6–10 in Fig 4A). The shear stress experienced by the cell and the time of exposure to that shear depend on the trajectory. This explains the variety in the rotation dynamics for the different trajectories.

As stated in [42, 44, 63], the shape factor *f*_*s*_ depends on the particle orientation. Hence, changes in *θ* should impact the electrical pulses. In Fig 4B, the electrical signatures are shown according to the axial coordinate *x* to ease the comparison with the views of Fig 4A. Regarding cases 5–10, Fig 4A and 4B show that the cell rotation induces a peak on the electrical pulse. By the term ‘peak’, we refer to an increase in Δ*R*, that is shorter than the pulse. As expected [63], the pulses maximum is reached at the exact moment when the cell is perpendicular to the aperture axis (*θ* = $\frac{\pi }{2}$) for cases 6–10. Despite the rotation observed for case 5 in Fig 4A, the pulse maximum does not match with the instant at which *θ* = $\frac{\pi }{2}$ (see case 5 in Fig 4B). This is because the RBC achieves a $\frac{\pi }{2}$ orientation outside of the orifice, where the electrical field rapidly decreases. An interesting result is that the closer the particle path to the wall, the earlier the rotation and the peak.

In summary, the numerical results highlight different RBCs dynamics inside the micro-aperture. High shear levels are present in the wall vicinity, which makes the RBC rotate when flowing near the wall. On the contrary, RBCs are perfectly aligned with the aperture axis when they flow along the centerline. In addition, the distance from the aperture wall drives the time spent by the cell in the detection area and the rotation rate: the closer the particle path to the aperture wall, the longer the time spent in the orifice and the earlier the rotation. These dynamical effects are visible on the generated pulses. In the following, experimental data are compared with the simulations in order to confirm these numerical observations. To allow this comparison, three features are introduced in Sec 3.2, for assessing the most relevant pulse characteristics. The comparisons, based on the later features, are presented in Sec. 3.3.

### 3.2 Relevant pulse features

**Fig 5:**
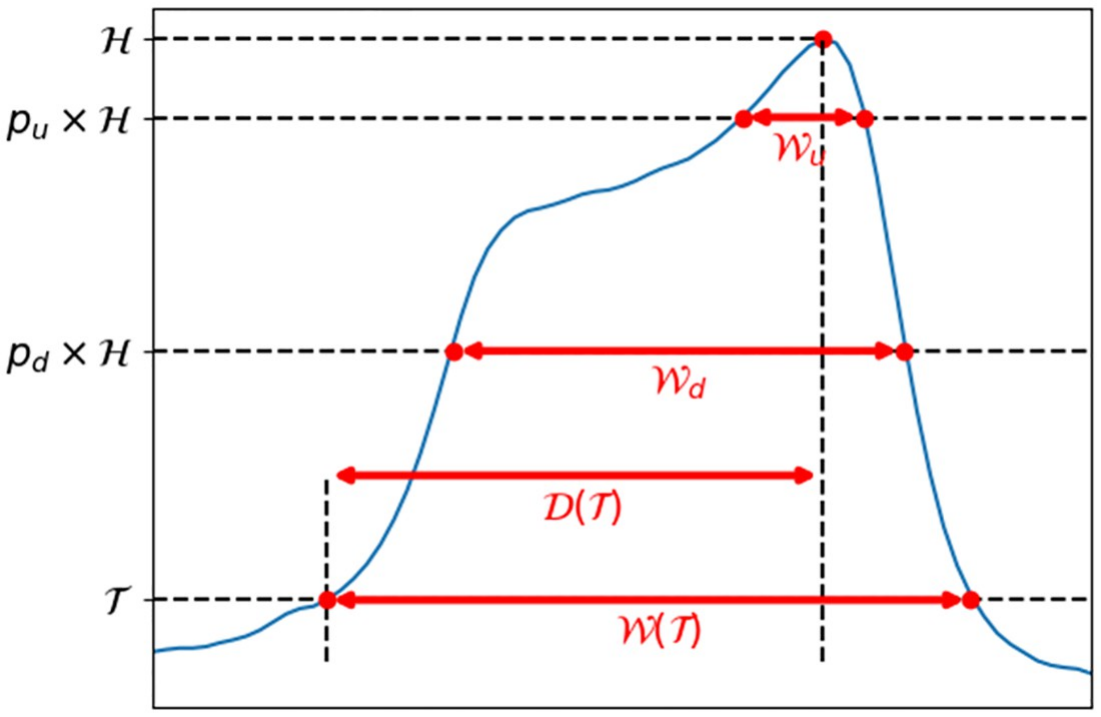
Illustration of quantities derived from impedance pulses and required for calculating features W, R and P.

We now define some features of the pulses, relevant to our problem. The pulse duration or pulse width W, calculated at a threshold T (as illustrated in Fig 5) quantifies the time spent by the cell in the aperture. The peak location, which is related to the moment at which the cellular rotation occurs, is assessed by the metric P:

$$\begin{array}{c} P\left(T\right)=\frac{D(T)}{W(T)}\times 100, \end{array}$$ (4)

with D the duration between the time when the signal exceed threshold T and the time when the maximum H is reached (see Fig 5). Hence, P(T) consists in assessing the relative time when the pulse maximum H is reached along W(T). The metric P is included in [0, 100] and increases as H is delayed on the pulse. It should be noted that both W and P depend on the threshold T. T should intersect the ascending and descending slopes of the signal for ensuring relevant results.

A feature built as the ratio of two pulse widths (with two different thresholds) was shown to detect whether a rotation-associated peak is observable on the pulse [43]. These two pulse widths, noted $W_{u}$ and $W_{d}$, are calculated at different thresholds: $H\times p_{u}$ and $H\times p_{d}$ (see Fig 5). The quantities *p*_*u*_ and *p*_*d*_ are parameters included in [0; 1], while H denotes the pulse maximum (see Fig 5). In this respect, the so-called ‘widths ratio’ R, writes:

$$\begin{array}{c} R=\frac{W_{u}}{W_{d}}\times 100 \end{array}$$ (5)

The threshold $H\times p_{u}$, for computing the width in the numerator of Eq 5, is designed to intersect the rotation-associated peak, if present. In contrast, *p*_*d*_ is set in such a way that $H\times p_{d}$ crosses the ascending and descending slopes of the pulses, so that $W_{d}$ quantifies the time spent by the cell in the aperture. Parameters *p*_*d*_ and *p*_*u*_ are set to 1/2 and 7/8 in the following. In this respect, pulses presenting a clear rotation-associated peak lead to low R values, whereas signatures without a rotation peak lead to R values typically higher than 50 [43].

### 3.3 Comparisons with experimental data

Experimental pulses were recorded during the analysis of a blood sample withdrawn from a healthy donor, as stipulated in Sec. 2. As raw experimental data are the result of an electrical signal amplification, direct comparison of the pulses is not possible. Simulations performed by varying the RBC volume have shown marginal effects on the pulse shape (results not shown). Hence, we focus on the comparison of non-dimensional pulses: the experimental measurements are scaled with the averaged maximum of the ‘bell-shaped’ pulses, which is a good measure of the averaged RBC volume, the MCV. ‘Bell-shaped’ pulses are generated by centered trajectories. Simple methods for extracting ‘bell-shaped’ signatures from an entire acquisition have previously been introduced [43]. For the non-dimensionalization of the numerical pulses, the scaling factor is the maximum of case 1, that yields a ‘bell-shape’ pulse (see Fig 3B). In the following, we denote by ΔU* and ΔR*, the scaled experimental voltage pulses and the scaled numerical resistive pulses, respectively. Note that W and P are calculated from the scaled pulses ΔU* and ΔR* with a dimensionless threshold T of 0.5.

**Fig 6:**
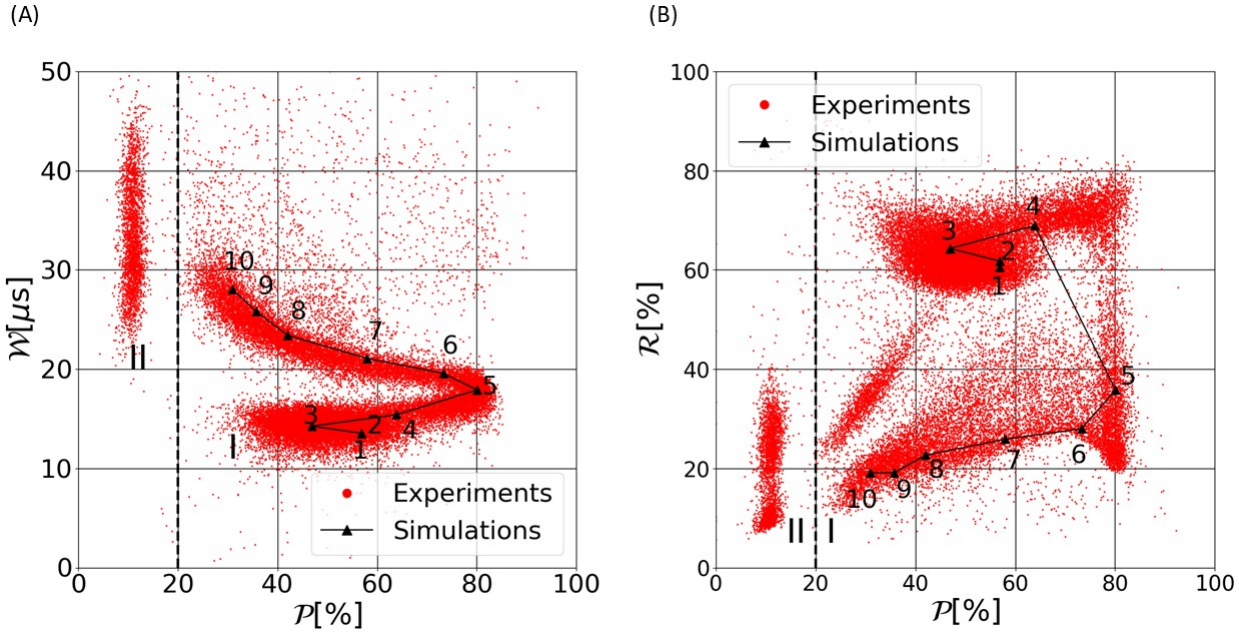
Scatter plots of experimental measurements and numerical results in the (W,P) plane (A) and in the (R,P) plane (B). Measurements from the experimental acquisition are depicted in red and the 10 numerical simulations with the reference parameters for the RBC and only changing the trajectory are represented by black triangles and linked with a black solid line to ease the visualization.

The scatter plot of W as a function of P is displayed in Fig 6A. The numbering in Fig 6 refers to the 10 simulated cases of Fig 3, in which only the trajectory changes. In this (W,P) plane, electrical pulses form two groups, labeled I and II in the figure. Group I gathers 94% of the pulses and group II, 6%. Group II is characterized by P = 10% ± 5 and a wide variety along W axis. Group I is organized in two main branches that join around P = 80% and W = 17 μs. The lower branch, for which W< 17 μs, is associated to cases 1 to 5 of the simulations. More precisely, it corresponds to the region of the aperture that extends from the perfectly centered path to the first trajectory on which the cell can reach a $\frac{\theta }{2}$ orientation inside the aperture (in between cases 5 and 6). The upper branch (W> 17 μs) corresponds to simulated trajectories 5–10 and displays a decreasing pulse duration (W) with respect to the peak position (P). This is in agreement with the previous statement that when the trajectory progressively approaches the aperture walls, the peak occurs sooner, and the pulse becomes longer. While group I is well predicted by the 10 simulations reported, group II is not. The pulses of group II feature a very early peak, which is consistent with a pulse maximum due to an electrical effect, the cell passing in a region where the electrical field is particularly high, at the corner of the orifice entrance. The associated electrical peak is clearly visible for the simulation of a rigid sphere case [42], for instance (shown later, in Fig 8C). Thus, pulses of group II may be explained by rounder cells and/or trajectories even closer to the orifice edges. This is supported by the large values of W measured in group II. Unfortunately, simulating such trajectories leads to unstable computations, and further developments are required to accurately reproduce this isolated cluster. Note also that some isolated experimental results show high values of W and P. They correspond to the passage of two particles at the same time.

Experiments and simulations are also compared in the (R,P) plane in Fig 6B. Data with R values below 50% are associated with pulses with a marked peak [43]. In contrast, measurements with R values higher than 50% arise from ‘bell-shaped’ pulses. They are generated by RBCs perfectly aligned with the aperture or which barely rotate in the aperture, such as cases 1–4 from the simulations (see Fig 3). As seen before, group II (P≈ 10%) does not correspond to any of our 10 simulations. In addition, another small cluster located at P≈ 30% and R≈ 30% is not recovered. Actually, pulses belonging to this cluster are similar to those superimposed with cases 8 to 10. The electrical field at the corner of the aperture, induces an electrical peak at the early beginning of the pulse. In some cases, this electrical peak adds up to the rotation peak, which skew the computation of R. Tuning the parameter *p*_*u*_ would alleviate this issue. Nevertheless, numerical results align well with the experimental acquisition and the pulses which are not reproduced by the simulation data set represents less than 6% of the RBC population.

**Fig 7:**
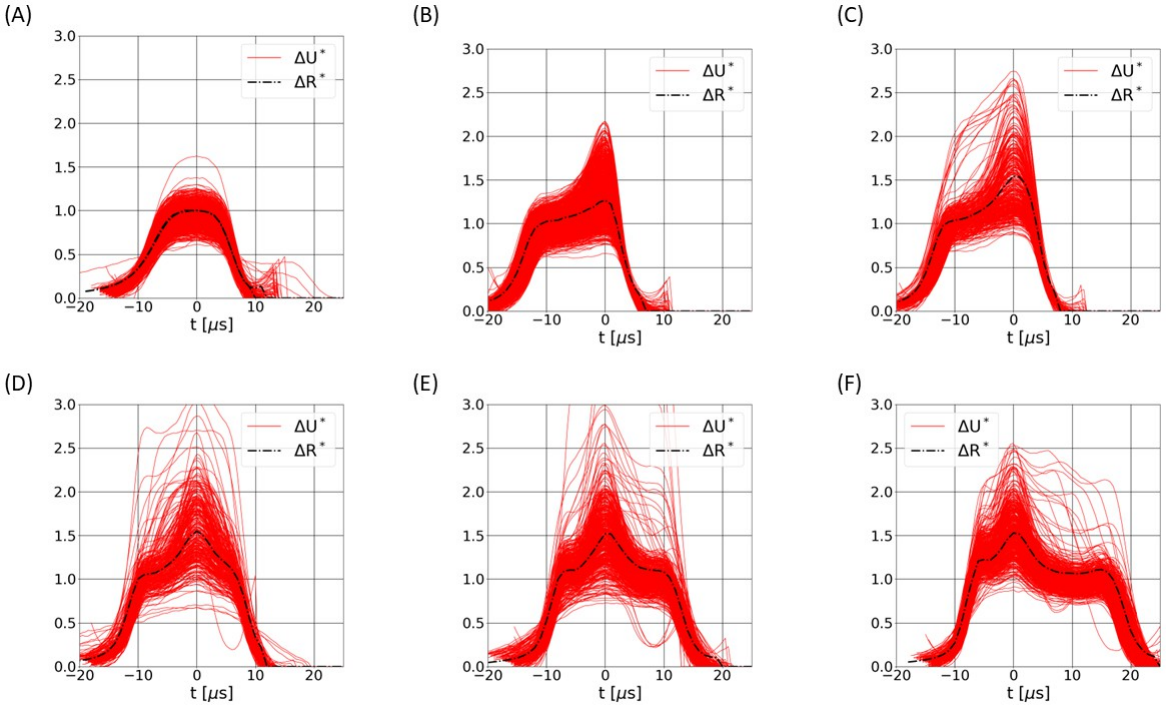
Comparison of the simulated pulses with experimental measurements. The numerical pulses are superimposed with experimental data (in red continuous line) that have the same W and P, with a tolerance margin of ±1 μs and ±2% respectively. Graphs A, B, C, D, E and F correspond to the simulated cases 1, 5, 6, 7, 8 and 10, respectively.

The 3 features W, R and P allow the summary of a time signal by 3 numbers. For a more detailed comparison, we compare the pulses having similar values of W and P. Let us define $P_{n}$ and $W_{n}$, the values of P and W, respectively, computed for a simulated pulse, and $P_{e}$ and $W_{e}$ the same metrics for an experimental pulse. In Fig 7, 6 numerical pulses are displayed and compared with experimental measurements that satisfy the two conditions: $P_{n}$—2% $<P_{e}<$ $P_{n}$ + 2% and $W_{n}$—1 μs $<W_{e}<W_{n}$ + 1 μs. Note that pulses are superimposed in such a way that their maximum occurs at time t = 0. The numerical predictions are in good agreement with the experiments, despite the variable amplitudes that originates from the discrepancy of RBC volumes within a blood sample, but also due to variable shape and rheological parameters of RBCs. Some experimental pulses exhibit shapes that do not correspond to numerical results. However, these cases are rare and can be explained by particle coincidences in the aperture.

## 4 Pulse dependency on the RBCs rheology

**Fig 8:**
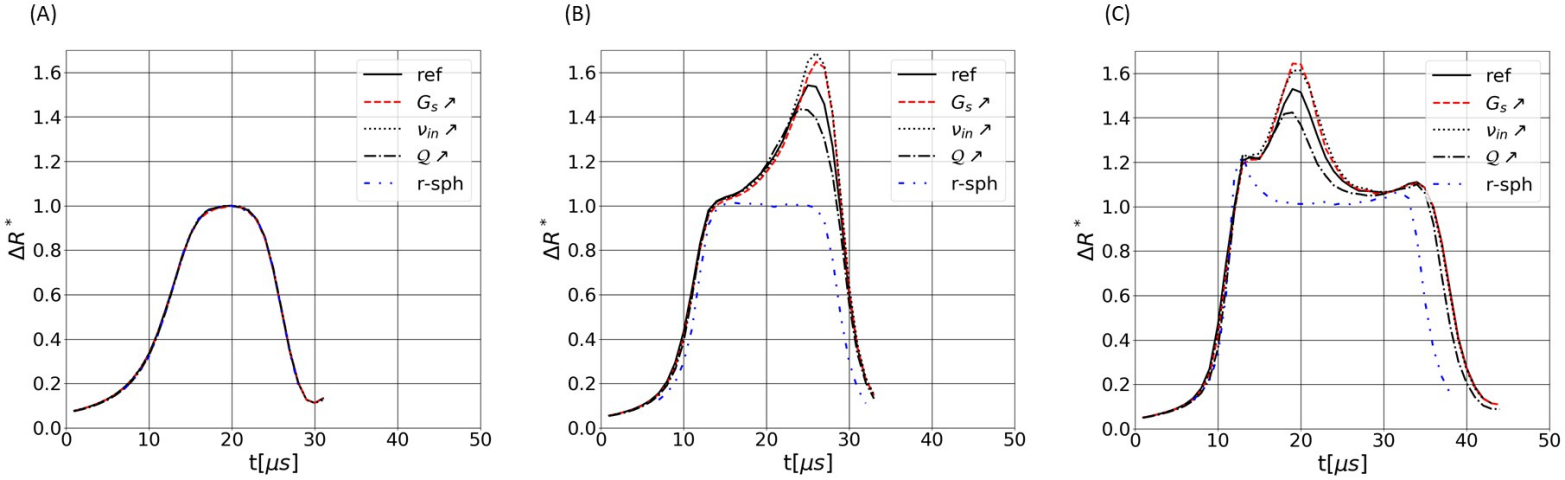
One-at-a-time sensitivity analysis of the effect of the shear modulus *G*_*s*_, the reduced volume Q and the internal viscosity *ν*_*in*_. Pulses of pictures A, B and C correspond to trajectories 1, 6 and 10 of Fig 3, respectively. The settings for the different depicted cases is given in Table 1.

The effect of the RBC rheology on the pulses is now investigated. Simulations over different trajectories are performed by increasing the shear modulus (*G*_*s*_), the internal viscosity (*ν*_*in*_) and the reduced volume (Q) of the RBC, one at a time. Fig 8 displays the numerical pulses for trajectories 1 (Fig 8A), 6 (Fig 8B) and 10 (Fig 8C), for the ‘ref’ series, also shown in Fig 3B, and 4 additional series with modified particle properties, summarized in Table 1. Regarding the central trajectory (see Fig 8A), the electrical signature does not depend on variations of the RBC parameters. However, cases 6 and 10 are found to vary with the cell features, as shown in Fig 8B and 8C. The internal viscosity has a similar impact as the membrane rigidity, since increases of *ν*_*in*_ and *G*_*s*_ both yield pulses with a more important peak. The cytosol viscosity reduces the instantaneous deformability of the cell. Hence, because of the short loading times experienced by RBCs in the aperture, an increase of viscosity yields a smaller RBC deformation inside the sensing region. As previously shown [42], a strong compression of the cell occurs while it rotates, which appears to mitigate the peak amplitude. Consequently, increases of *ν*_*in*_ and *G*_*s*_ make the RBC harder to compress and produce a larger peak. The amplitude of the peak is a decreasing function of the reduced volume (Q), contrary to *ν*_*in*_ and *G*_*s*_. A higher reduced volume implies a more spherical cell, which tends to conceal the consequences of the cell rotation (viz. the peak). Results for rigid spheres are also presented in Fig 8 and confirm this observation, since no rotation-associated peaks are observed for pulses referred as ‘r-sph’. The settings employed for handling rigid spheres in the simulations is given in Table 1.

**Table 1:** Summary of the parameters used in the simulations performed to study the effect of shape and rheology of particles on the pulses. Each column corresponds to a series of simulations, for different trajectories. Cases ‘ref’, ‘*G*_*s*_↗’, ‘*ν*_*in*_↗’ and ‘Q↗’ are relevant for RBCs, while case ‘r-sph’ models a rigid sphere.

	ref	*G*_*s*_↗	*ν*_*in*_↗	Q↗	r-sph
*G*_*s*_[N.m^−1^]	2.5×10^−6^	40.0×10^−6^	2.5×10^−6^	2.5×10^−6^	2.5×10^−3^
*ν*_*in*_[m^2^.s^−1^]	18×10^−6^	18×10^−6^	21×10^−6^	18×10^−6^	50×10^−6^
Q	0.65	0.65	0.65	0.75	1.0

**Fig 9:**
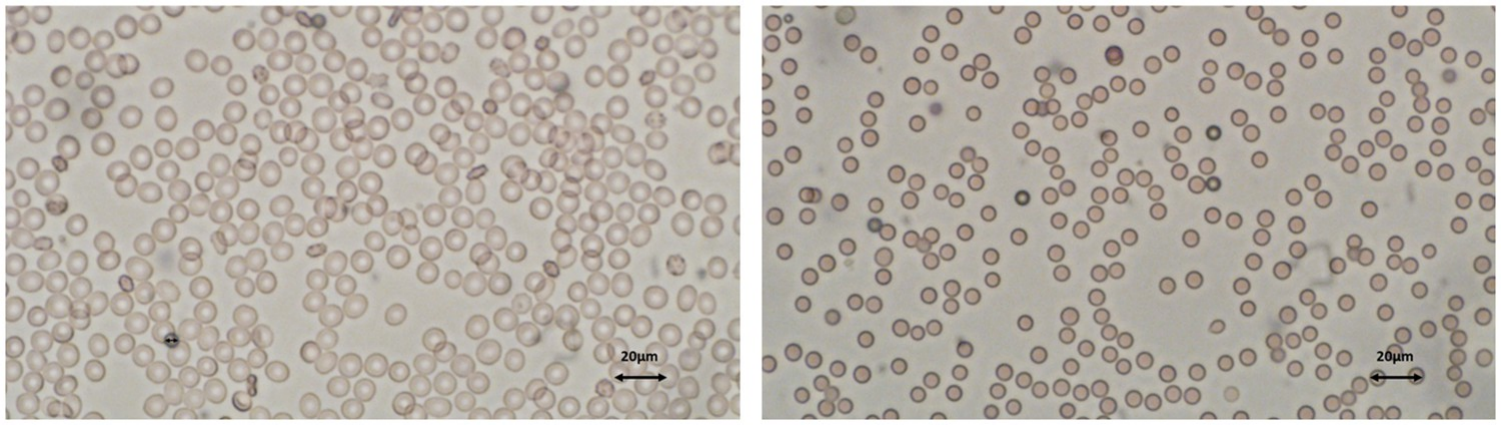
Microscopic views of RBCs suspended in the HORIBA Medical electrolytic reagent (left picture) and in a SB3–12 solution at 100 mg.L^−1^ concentration (right picture).

In order to validate the tendencies observed in Fig 8, experiments were performed with spherized and stiffened RBCs, respectively. In these experiments, the alteration of RBCs properties is allowed by two distinct agents: the glutaraldehyde, which is known to fix the RBCs membrane [54], and a surfactant, the n-Dodecyl-N, N-dimethyl-3-ammonio-1-propanesulfonate also called sulfobetaine 3–12, noted here SB3–12 (provided by Sigma-Aldrich), that is shown to make the RBCs spherical. Glutaraldehyde is a molecule that crosslinks the RBC proteins, thus stiffening the cell. Indeed, several studies reporting a reduction of the RBCs deformability when they are treated with glutaraldehyde are available in the literature [64, 65]. The discocyte shape of RBCs is maintained at glutaraldehyde concentration below 1% [66]. Contrary to glutaraldehyde, SB3–12 has not been extensively studied. Experiments revealed that RBCs are spherical at SB3–12 concentrations of about 100 mg.L^−1^ (see Fig 9). In addition, such changes appear to produce rapidly, since all cells are round 20 s after being treated with SB3–12.

Several dilutions of SB3–12 and glutaraldehyde in ABX Minidil LMG (HORIBA Medical) are prepared for the experimental acquisitions. In particular, SB3–12 dilutions cover a concentration range in between 0 and 90 mg.L^−1^, while glutaraldehyde is diluted at concentrations included in [0%, 0.5%]. Then, pulses acquisitions are performed with an ABX Micros 60, as usual, but replacing the classical reagent (ABX Minidil LMG) with the modified solutions. On the ABX Micros 60, the replacement of the electrolytic solution is done by interchanging the reactant bottles. Besides, the ‘CLEAN ALL REAGENT’ command is run for removing the former solution from the needle and the system pipes.

**Fig 10:**
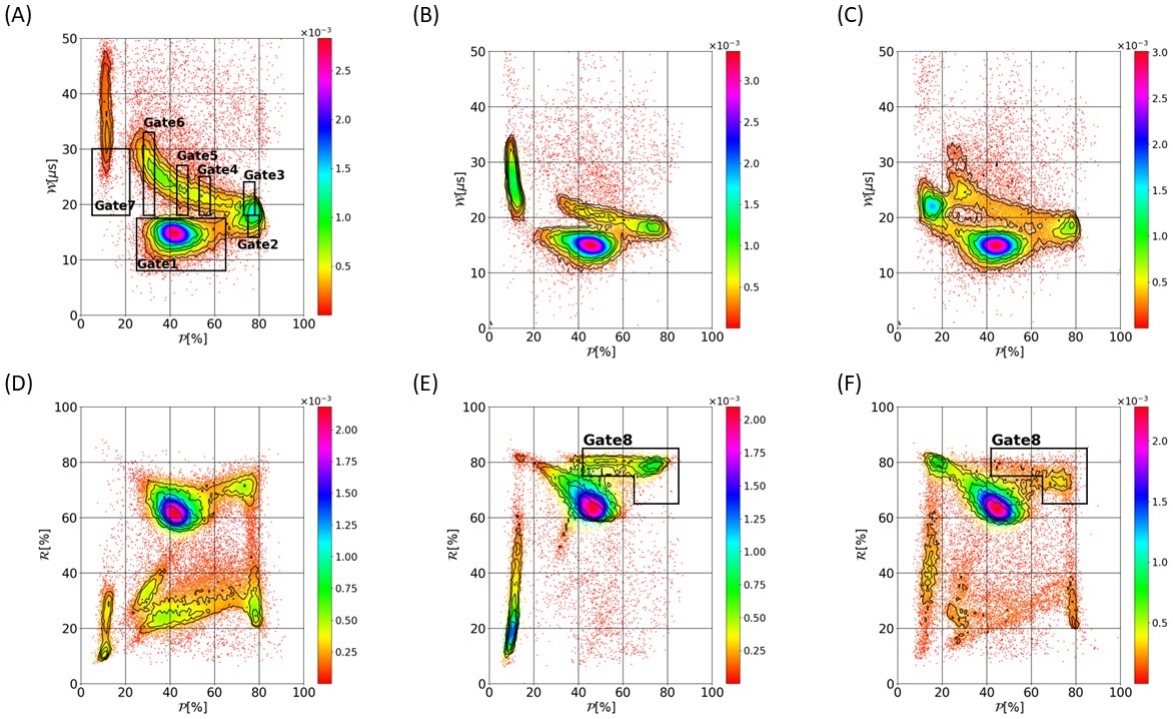
Dependence of (W,P) and (R,P) density maps to the morpho-mechanical characteristics of the cells. Graph A and D are obtained without SB3–12 nor glutaraldehyde. Pictures B and E arise from the acquisition with 90 mg.L^−1^ SB3–12, while graphs C and F are derived from the acquisition with 0.5% glutaraldehyde. In pictures A, E and F, the gating used for the extraction of pulse signatures (Gates 1–6) and for assessing RBCs alteration (Gate 7 and 8) is shown. The colormaps derive from Gaussian kernel density estimations, while the black lines are density isolines derived from the actual distributions.

**Fig 11:**
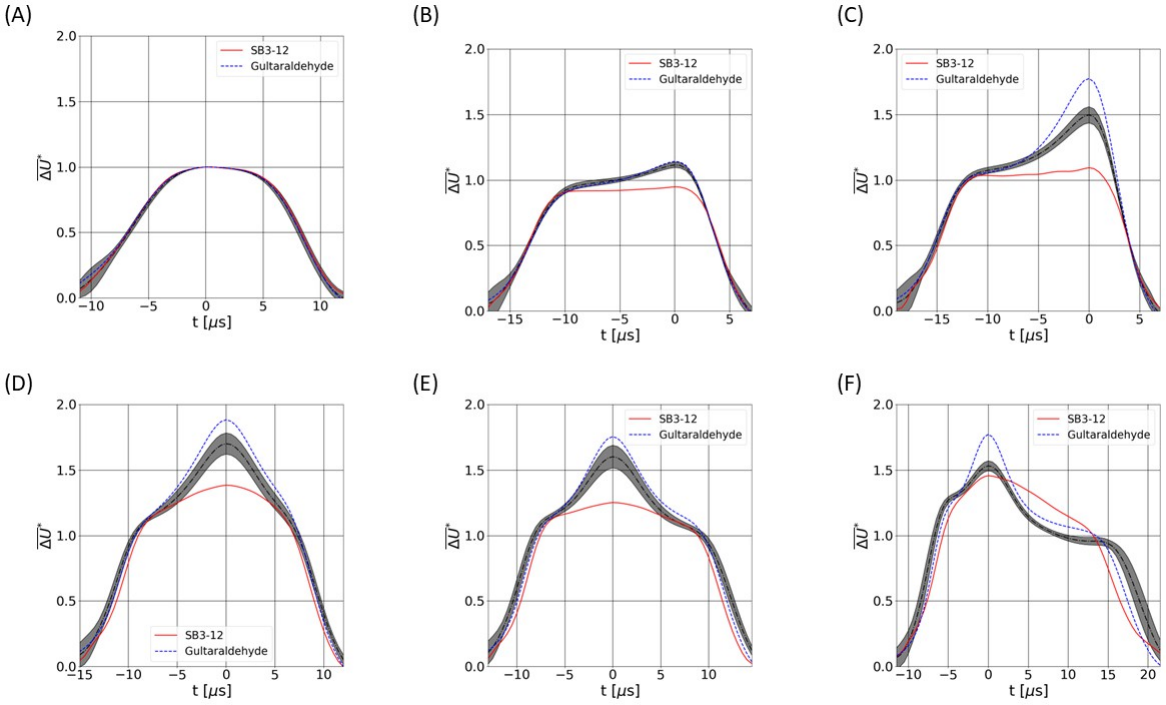
Gate-wise comparison of the averaged pulses signatures. Pictures A, B, C, D, E and F correspond to Gates 1, 2, 3, 4, 5 and 6 (see Fig 10), respectively. The grey area illustrates the confidence intervals (±2*σ*) derived from 22 samples withdrawn from healthy donors. Glutaraldehyde concentration of 0.5% and SB3–12 concentration of 90 mg.L^−1^ have been used.

Fig 10 displays typical (W,P) and (R,P) density plots arising from acquisitions without treatment (Fig 10A and 10D) and with treatment, with the maximum concentrations of SB3–12 (Fig 10B and 10E) and glutaraldehyde (Fig 10C and 10F) of our ranges, to highlight the effect of the drugs. Fig 10A is similar to Fig 6A, with results displayed as a density map instead of a scatter plot. Adding glutaraldehyde or SB3–12 is found to impact the distribution and location of the pulses on both (W,P) and (R,P) maps, as Fig 10 illustrates. In order to assess the impact of the drugs on the pulses themselves, we select specific regions of the (W,P) plane to compare the pulses having similar values of W and P: those regions are defined by so-called ‘Gates’ in Fig 10A and correspond to different trajectories (Gate 1 is for central trajectories for instance). For each of the Gates 1 to 6, an average pulse is calculated for the 2 drug treatments. In Fig 11, those averaged signatures are compared in a gate-by-gate manner with results coming from the analysis of 22 samples in normal conditions (viz. without SB3–12 nor glutaraldehyde). Regarding Gate1, related to centered trajectories, no deviation is observed in terms of averaged pulses (see Fig 11A). Stiffening RBCs with glutaraldehyde tends to increase the magnitude of the peak associated with cell rotation (see case ‘Glutaraldehyde’ in Fig 11C–11F). This is consistent with the numerical results of Sec. 3.2 obtained with less deformable cells. Contrary to glutaraldehyde, SB3–12 is found to reduce the magnitude of the rotation-associated peak (see Fig 11B–11F): as expected, the signature of the cellular rotation is made less visible by the spherization of the RBCs due to the SB3–12 treatment. Again, simulations performed with an increased reduced volume Q (thus, cell sphericity) have shown the same trend (see Fig 8).

In summary, the morpho-mechanical properties of RBCs are shown to impact the electrical pulses, with both numerical and experimental approaches. In addition, the pulses distributions on (W,P) and (R,P) planes are found to vary with the RBCs properties. In the following section, we present a proof of concept for the detection of RBCs disorders from the pulse’s distributions on (W,P) and (R,P) graphs.

## 5 Towards the detection of RBCs disorders

**Fig 12:**
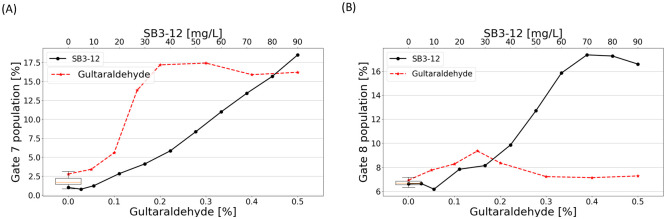
Variations of the pulse proportion inside Gate 7 and 8 (see Fig 10) as a function of glutaraldehyde and SB3–12 concentrations. The boxplots arise from measurements from 22 healthy donors, without glutaraldehyde nor SB3–12.

The cluster located at P<20% (viz. group II in Fig 6) is substantially impacted by the morpho-mechanical alterations of RBCs. Indeed, this cluster is found to shift towards low values of W, when considering the experiments with glutaraldehyde and SB3–12 (see Fig 10). This can be explained by the reduced deformability of erythrocytes when stiffening their membrane or spherizing the cell. Indeed, for trajectories very close to the aperture walls, this would make the cell unable to deform on the orifice corner and deflect the cell through the center, thus reducing the pulse duration W because of the higher velocity near the orifice axis. This was illustrated by considering rigid spheres in the simulations (see Fig 8C). The sphere starting from the same position upstream of the orifice is deflected toward the aperture axis [42], which leads to a shorter pulse (see case ‘r-sph’ in Fig 8C). In this respect, Gate 7 is defined to correspond to cells passing very close to the wall (with a high electrical peak associated with a low value of P), but low values of pulse duration W compared with the normal case. In Fig 12, the percentage of pulses inside Gate 7 is displayed as a function of SB3–12 and glutaraldehyde concentrations. As expected, the proportion increases with both glutaraldehyde and SB3–12. This result shows that the effect of drug treatment is gradual, which means that the number of cells in Gate 7 is an indication of the average properties of RBCs in the sample.

While Gate 7 was introduced for assessing deformability alterations of cells, Gate 8 (depicted in Fig 10E and 10F) is designed to shed light on shape alterations of RBCs. When spherizing the cells, the rotation-associated peaks are limited. For that reason, $H\times p_{u}$ may not intersect the peak, thus leading to high R values, even if the cells have followed a near-wall trajectory. This explains why the population inside Gate 8 becomes denser when spherizing RBCs with SB3–12, whereas it remains at a reasonable level with glutaraldehyde, as Fig 10E and 10F illustrate. This is confirmed by Fig 12B, where the percentage of pulse inside Gate 8 is shown as a function of SB3–12 and glutaraldehyde concentrations. The proportion substantially increases with the SB3–12 concentration (from 6.6% to 17.3%), while it remains below 9.4% when cells are submitted to glutaraldehyde. It should be noted that differentiating the two types of disorder is impossible by assessing solely the population inside Gate 7, since glutaraldehyde and SB3–12 were both found to increase the percentage of events. However, if the cells are sufficiently impacted, the distinction may become possible by considering both Gates 7 and 8.

## 6 Conclusions

New Insights on the dynamics of RBCs in Coulter-based systems are provided, relying on a numerical approach. Depending on the trajectory, the cell is subjected to different shear levels and exposure times, thus leading to a variety of rotation dynamics in the sensing region. The closer the RBCs path to the aperture walls, the longer the cell stays in the sensor and the sooner the rotation occurs. In terms of electrical pulses, this translates into longer signals with an earlier peak, which has been shown to occur when the cell rotates. Hence, three metrics are introduced for assessing the most relevant pulse features: W the pulse duration, P the peak location and R the ratio of two pulse widths, which indicates weather a marked cellular rotation has occurred. Good agreements between numerical results and experimental data are found in terms of (W,P) and (R,P) scatter plots (viz. at the scale of the whole sample) and in terms of detailed pulses shape. This not only validates the numerical results, but also explains the variety of pulse signatures observed experimentally.

A part of experimental acquisitions was not retrieved in the simulations. This portion, representing approximately 6% of an entire acquisition, is isolated from the remaining part on (W,P) and (R,P) graphs. More precisely, it is located at P below 20% and W between 25 μs and 45 μs. Considering trajectories closer to the aperture edges than that simulated in this work would likely explain this pulse population. Indeed, the aforementioned pulses are longer (viz. higher W) than the rest of the acquisition. Furthermore, at the corner of the orifice, the electrical field is dense, which generates an electrical peak very early in the pulse. For trajectories really close to the edges, the electrical peak may be greater than the rotation associated peak, which could explain why these pulses are detached from the remainder along the peak position (P) axis. Unfortunately, numerical instabilities are encountered in such wall proximity and make difficult any conclusions. Hence, further numerical efforts are intended in the future to reproduce accurately this type of signatures.

The range of shear rate experienced by the cell in commercial Coulter-based systems is much higher than that of the configurations generally studied in the literature. Nevertheless, RBC deformations remain moderate. The RBC exposure times to these high shear rates being very short, the internal viscosity plays an important role in maintaining the cell shape. The same should be true for the membrane viscosity, although it is not modeled in the numerical simulation. Dielectrophoretic forces acting on the membrane were not taken into account, while RBC electro-deformations were reported [67, 68], in the range of electrical field observed in the detection area. Further investigation about the effect of the membrane viscosity [57] and the DEP forces should be performed in the future.

The impact of the RBC parameters on the measured pulse has been investigated. In particular, when increasing the membrane rigidity and the cytosol viscosity in the simulations, the rotation-associated peak is found to increase. On the contrary, a more spherical RBC is shown to decrease the amplitude of the peak. These numerical results are confirmed by experimental pulses acquisitions performed with spherized and stiffened RBCs. In these experiments, rigidified and spherized erythrocytes are obtained by adding glutaraldehyde and SB3–12 in the electrolytic solution, respectively.

The pulses repartition on (W,P) and (R,P) graphs is shown to vary when altering the RBCs properties. In particular, some regions of the aforementioned maps, usually unpopulated, may become substantially dense. In this respect, original markers which consist in assessing subpopulations proportions from (W,P) and (R,P) graphs are introduced and shown to highlight RBCs alterations. Differentiating the two types of alteration considered in this work appears to be feasible by combining information arising from both graphs. It should be noted that the gating employed for the definition of the subpopulations has not been optimized. Furthermore, in this proof of concept, only artificial alterations of RBCs were considered. Testing the methods on true RBCs pathologies will be done in the near future, for assessing the relevance of the original markers for clinical purposes. Finally, although the proposed markers provide a straightforward detection of abnormalities, they are limited to pathologies affecting a substantial proportion of RBCs. Nevertheless, this work demonstrates the possility of using already commercialized hematology analyzers to assess the shape and rheological properties of RBCs at high-throughput, opening the way to a more complete RBC diagnosis in the near future.

## Supporting information

S1 File Experimental dataset. The experimental acquisitions are available at the following DOI: https://doi.org/10.6084/m9.figshare.21901590.v1.
(TXT)
